# Properties of Polylactic Acid Reinforced by Hydroxyapatite Modified Nanocellulose

**DOI:** 10.3390/polym11061009

**Published:** 2019-06-06

**Authors:** Jianxiao Lu, Chuanyue Sun, Kexin Yang, Kaili Wang, Yingyi Jiang, Rogers Tusiime, Yun Yang, Fan Fan, Zeyu Sun, Yong Liu, Hui Zhang, Keqing Han, Muhuo Yu

**Affiliations:** 1 State Key Laboratory for Modification of Chemical Fibers and Polymer Materials, College of Materials Science and Engineering, Donghua University, Shanghai 201620, China; ljxstayreal@foxmail.com (J.L.); woai82dian@protonmail.com (C.S.); kexin_yang@163.com (K.Y.); 18616146552@163.com (K.W.); 13888665350@163.com (Y.J.); 317001@mail.dhu.edu.cn (R.T.); 2180462@mail.dhu.edu.cn (Y.Y.); 18256787163@163.com (F.F.); sunzeyu@dhu.edu.cn (Z.S.); liuyong@dhu.edu.cn (Y.L.); hankeqing@dhu.edu.cn (K.H.); 2 Shanghai Key Laboratory of Lightweight Structural Composites, Shanghai 201620, China

**Keywords:** nanocellulose, hydroxyapatite, polylactic acid, compatibility

## Abstract

Polylactic acid (PLA) is one of the most promising bio-based materials, but its inherent hydrophobicity limits its application. Although nanocellulose (NCC) is a desirable reinforcement for PLA, the poor interface compatibility between the two has been a challenge. In this work, hydroxyapatite (HAP) modified NCC was prepared, and the obtained NCC/HAP reinforcement was used to prepare PLA/NCC-HAP composites. Different ratios of NCC to HAP were studied to explore their effects on the mechanical and thermodynamic properties of the composites. When the ratio of NCC to HAP was 30/70, the tensile strength and tensile modulus of the composite film reached 45.6 MPa and 2.34 GPa, respectively. Thermogravimetric analysis results indicate that thermal stability of the composites was significantly improved compared with pure PLA, reaching 346.6 °C. The above revelations show that NCC/HAP significantly improved the interface compatibility with PLA matrix.

## 1. Introduction

Since polylactic acid (PLA) was approved by the United States Food and Drug Administration (FDA) in 1970, PLA has emerged as one of the most promising biopolymers. Nowadays, numerous research efforts have been dedicated to developing environmentally-friendly materials to replace the original petroleum-based materials and expand various biomedical applications, like drug delivery and tissue engineering [1,2,3]. Polylactic acid has the advantages of biocompatibility and biodegradability, high strength, high modulus and good optical transparency [4]. Its applications include, but are not limited to, medical, fiber and textiles, packaging, plastic cultivation, environmental restoration, paint, cigarette filters, 3D printing and space exploration parts. However, polylactic acid has obvious disadvantages such as inherent hydrophobicity, low crystallinity and poor heat resistance, which hinders its extensive application and development [5]. Blending is a simple and effective way to modify the properties of polymers [6,7,8]. The use of fibers or fillers to enhance properties is the primary means of improving performance of polylactic acid [9]. Among the fibers or fillers, nanocellulose (NCC) is a biocompatible and biodegradable biopolymer, which has become one of the most popular reinforcing materials for polylactic acid.

Cellulose is the most abundant biomass material in nature and derives from corn, paddy crops, natural vegetation, algae and bacteria. Extracted from cellulose, nanocellulose and nanocellulose-derived materials are variously available, like film, building materials, medicines and food, due to its hydrophilicity, biodegradability and chemical modification ability [10,11,12,13,14,15]. Now, the nitro-oxidation method is a common method to prepare nanocellulose, and the water purification treatment is a popular application [16,17,18,19]. Also, based on the special physicochemical properties of nanomaterials, nanocellulose is a promising reinforced material for polylactic acid to improve the mechanical properties [20]. However, it is still a challenge to uniformly distribute the nanocellulose in polylactic acid matrix because of the strong hydrophilicity of nanocellulose and the hydrophobicity of polylactic acid. Research on the physical and chemical modification of nanocellulose to enhance the interfacial compatibility between nanocellulose and polylactic acid has been reported [21,22,23,24,25,26]. However, the treatment of inorganic particles and organic particle fillers still accounts for the majority [27,28,29,30,31,32].

The treatment of inorganic particle to obtain nanocellulose/inorganic particle composites is one of the most commonly used methods for nanocellulose modification [33]. Mahmoudian et al. [34] used a wet spinning method to prepare a regenerated cellulose/montmorillonite nanocomposite fiber with an environmentally friendly ionic liquid. The results showed that when the mass fraction of montmorillonite was 6%, the composite had good dispersion, and the thermal stability and mechanical properties of the composite were improved. Garusinghe et al. [35] developed a novel composite material that disperses montmorillonite into nanocellulose and achieves low water vapor permeability. However, the compatibility of montmorillonite was so poor that the prepared composites cannot meet the practical application requirements. Also, the modification of montmorillonite still had many complications such as high price, large dosage, intricate modification process and low efficiency. The same handicaps were observed with carbon nanotubes [36] and graphene oxide [37].

Hokkanen et al. [38] prepared a hydroxyapatite-bentonite-clay-nanocellulose composite as an adsorbent to remove Ni^2+^, Cd^2+^ and PO_4_^3−^ from aqueous solutions. Niamsap et al. [39] synthesized bacterial nanocellulose/hydroxyapatite/cellulose nanocrystal composites which can be used in tissue scaffolds in the medical field because of the biocompatibility. The above works used hydroxyapatite (HAP) to prepare nanocellulose composites, expanding the application of the composites. However, there was no research regarding the influence of different ratios of NCC to HAP on properties of the composites.

PLA has inherent hydrophobicity, while both NCC and HAP are hydrophilic. Herein, we hope to use the hydrophilicity of the two to reduce the hydrophobicity of PLA for more expanding application. In addition, due to biodegradability, biocompatibility and high strength, both NCC and HAP meet current environmental requirements and overcome the shortcomings of PLA such as, low crystallinity and heat resistance, improving mechanical properties [40]. For NCC, HAP and NCC/HAP nanofillers, the microscopic interface compatibility can be observed indirectly from macroscopic performance, such as tensile test. Only NCC and only HAP commonly need modification before enhancing PLA, while for NCC/HAP, itself is one method of modification. NCC is usually used to reinforce the PLA matrix. However, modification is necessary to coordinate the interfacial compatibility. In this work, NCC was modified by HAP and its surface is changed from smooth to rough, which increased the contact area with polymer matrix. When the NCC/HAP was blended with PLA, the NCC/HAP with a rough surface operated like an anchor to the PLA matrix, which contributed to the increase of the composite mechanical properties [41]. Therefore, NCC/HAP with different ratios was prepared to reinforce PLA. Properties of the obtained PLA/NCC-HAP composites were systematically investigated. The results show that NCC/HAP is a good reinforcing material for PLA, enhances the interfacial compatibility and improves the mechanical and thermodynamic properties of the composites.

## 2. Materials and Methods

### 2.1. Materials

Cotton pulp (DP: 350) was obtained from Jiangsu, China. Analytical reagent of sulfuric acid, sodium hydroxide, calcium nitrate tetrahydrate, diammonium phosphate, ethanol and chloroform were purchased from Sinopharm Chemical Reagent Co. Ltd., Shanghai, China. Polylactic acid (3052D) was produced by NatureWorks LLC, Blair, NE, USA. All the reagents were used as received without further purification.

### 2.2. Preparation of NCC

The cotton pulp was pulverized into cotton cellulose using a pulverizer. The cellulose and the sulfuric acid solution (64% by weight) were mixed at a ratio of 1:20 g/mL (cellulose: sulfuric acid solution) and stirred in a water bath at 50 °C for 1 h. The suspension was diluted 10-fold with deionized water to stop the reaction and stood for 24 h. The dilution was then centrifuged at 10,000 rpm multiple times to concentrate the cellulose and remove excess acid. The precipitated material was rinsed with distilled water and centrifuged again. Then the procedure repeated. Then sodium hydroxide was added to adjust the pH to 7. The neutralized suspension was sufficiently dispersed by an ultrasonic cell disrupter, and finally freeze-dried to obtain nanocellulose [42].

### 2.3. Preparation of NCC-HAP

**Figure 1:**
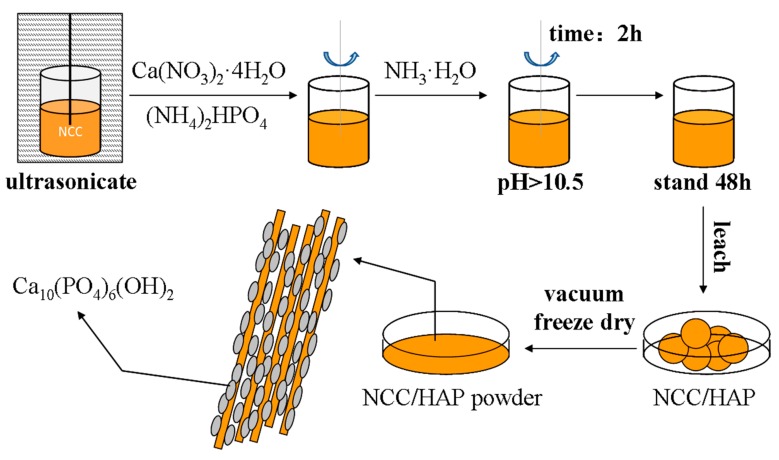
Schematic diagram of nanocellulose/hydroxyapatite (NCC/HAP) preparation.

An appropriate amount of nanocellulose was thoroughly dispersed in deionized water. Calcium nitrate tetrahydrate and diammonium hydrogen phosphate were added into the nanocellulose suspension, the mixture was stirred at 70 °C for 2 h, adding ammonia water to adjust the pH to above 10.5. After this, the resulting suspension stood for 48 h, it was filtered, and the obtained cake was washed with water and ethanol, respectively. The filtered cake was freeze-dried for 24 h, then ground to obtain NCC/HAP powder. A schematic diagram of NCC/HAP preparation is given in Figure 1. According to the above experimental procedure, NCC/HAP powders with different ratios were prepared respectively, as shown in Table 1.

### 2.4. Preparation of PLA/NCC-HAP

PLA and the NCC/HAP were each dried in a vacuum oven for 24 h. NCC/HAP (0.05 g) was then weighed and thoroughly dispersed under ultrasound in chloroform. PLA (5 g) was dissolved in chloroform by magnetic stirring. The dispersed NCC/HAP chloroform suspension was added into the PLA chloroform solution. The suspension solution for PLA/NCC-HAP system was sequentially ultrasonicated, stirred, poured into a glass garden, dried at room temperature for 24 h, then under vacuum at 40 °C for 12 h to obtain a PLA/NCC-HAP composite film. The composite film was subjected to a hot press forming at a temperature of 180 °C under a force of 500 kg for 8 min to obtain dumb bell-shaped PLA/NCC-HAP composite films. The prepared PLA/NCC-HAP composite films with different ratios of NCC to HAP are shown in Section 2.3. The additional content of NCC or NCC/HAP in composites was 1% wt of PLA.

### 2.5. Characterization

The morphologies of NCC and NCC/HAP were observed using a JEM-2100 Transmission Electron Microscope (TEM).

A Nicolet In10MX/Nicolet 6700 Fourier infrared (FT-IR) microscopy spectrometer was used to analyze the chemical structure of the samples. Prior to testing, the powdered sample was mixed and ground with potassium bromide and then compressed into tablets on an infrared tablet press.

X-ray diffraction (XRD) characterization was carried out on a D/max-2550VB+/PC type 18 KW target X-ray diffractometer. The test was performed using a CuKα (λ = 0.154 nm) light source, with diffraction angle 5–50° and a scanning rate 5°·min^−1^.

The tensile test of the dumb bell-shaped PLA/NCC-HAP composite films was determined with an Instron universal material testing machine. And the fractured surface of PLA/NCC-HAP was examined by using an S-4800 Field Emission Scanning Electron Microscope (SEM) coupled with Energy Dispersive Spectrometer (EDS).

Differential scanning calorimetry (DSC) of PLA/NCC-HAP samples was performed on a Q20 differential scanning calorimeter (conventional, of American TA Corporation). The test conditions were as follows: heating to 180 °C from 30 °C at a heating rate of 10 °C·min^−1^ under nitrogen atmosphere, holding for 5 min, then cooling to 30 °C at a rate of 10 °C·min^−1^, then raising to 180 °C at a rate of 10 °C·min^−1^.

The thermal stability of PLA/NCC-HAP samples was tested on a Q500IR Thermal Gravimetric Analyzer (TGA) of American TA Company. The samples were heated from 40 °C to 700 °C at a rate of 20 °C·min^−1^ under nitrogen atmosphere.

## 3. Results

### 3.1. Characterization of NCC/HAP

#### 3.1.1. Morphology of NCC/HAP

**Figure 2:**
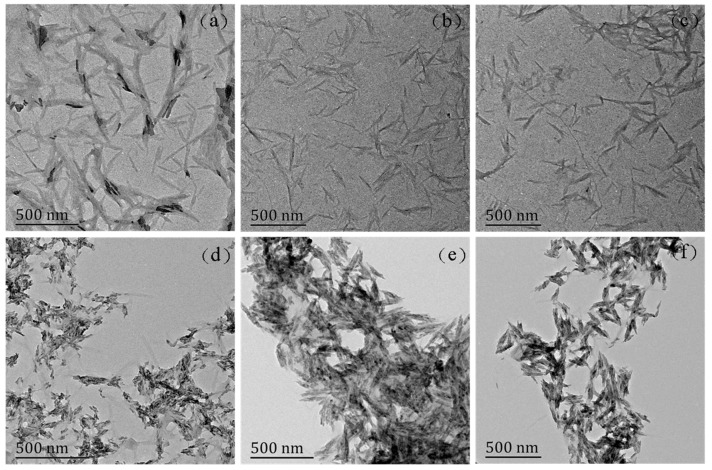
Transmission Electron Microscope (TEM) micrographs of NCC (**a**), NCC/HAP50/50 (**b**), NCC/HAP40/60 (**c**), NCC/HAP30/70 (**d**), NCC/HAP20/80 (**e**) and NCC/HAP10/90 (**f**).

In order to clarify the dispersity of the HAP in NCC, the surface morphologies of NCC and NCC/HAP were observed using TEM. The results are shown in Figure 2. Since NCC is one of the nanomaterials, it is easy to agglomerate in dispersion. However, it has been found that NCC still exhibited a rod-like structure. As can be seen from Figure 2a, the prepared NCC has a length of ca. 300 nm and a diameter of ~30 nm. Moreover, from the TEM images in Figure 2b–f, NCC/HAP with different ratios of NCC to HAP still maintained a rod-like structure. There were accumulated substances on the surface, which increased the diameter. These phenomena suggest that HAP disperses on the surface of the NCC. However, since the concentration of the dispersion was high when preparing samples for TEM testing, agglomeration occurred as seen in Figure 2b–f.

#### 3.1.2. FT-IR of NCC/HAP

**Figure 3:**
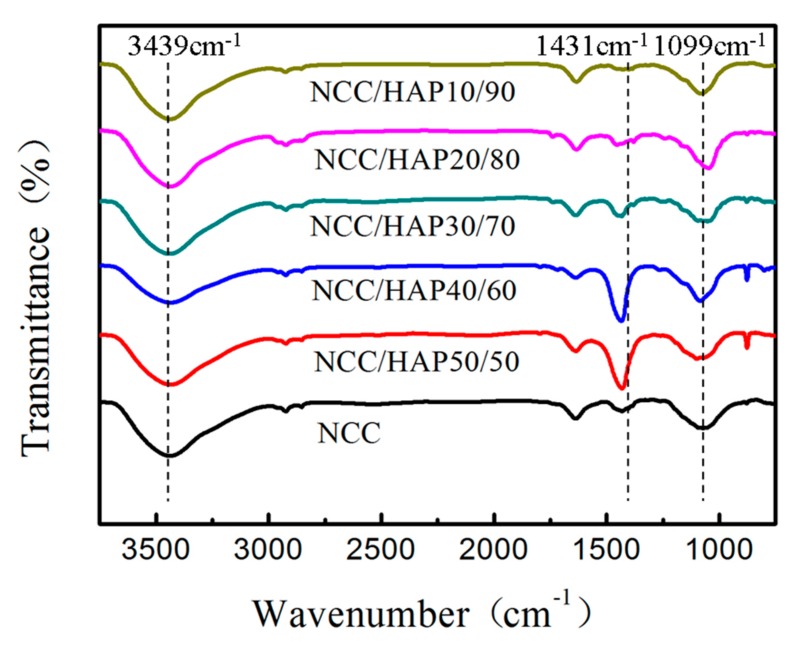
Fourier infrared (FT-IR) spectra of NCC, NCC/HAP50/50, NCC/HAP40/60, NCC/HAP30/70, NCC/HAP20/80 and NCC/HAP10/90.

Infrared spectroscopy was used to analyze the structural changes before and after nanocellulose modification. The infrared spectra of NCC and different proportions of NCC/HAP are shown in Figure 3. For NCC, the absorption peak of 3439 cm^−1^ is attributed to intramolecular hydrogen bond stretching and peak of 1630 cm^−1^ is assigned to O–H bending due to adsorption of water [43]. The peaks around 2900 cm^−1^ and 2850 cm^−1^ correspond to the –CH_2_ and –CH_3_ aliphatic groups. The absorption peak of 1431 cm^−1^ is attributed to CH_2_ shearing motion in cellulose and peak of 1099 cm^−1^ is assigned to stretching vibration of C–O [44,45]. It can be seen that NCC/HAP has above the same peaks as NCC, while the broad peak around 1100–1000 cm^−1^ also corresponds to phosphoric acid group PO_4_^3−^ stretching vibration. In addition, peaks at 877 cm^−1^ and 1420–1480 cm^−1^, correspond to the CO_3_^2−^ band [46].

#### 3.1.3. X-Ray Diffraction of NCC/HAP

**Figure 4:**
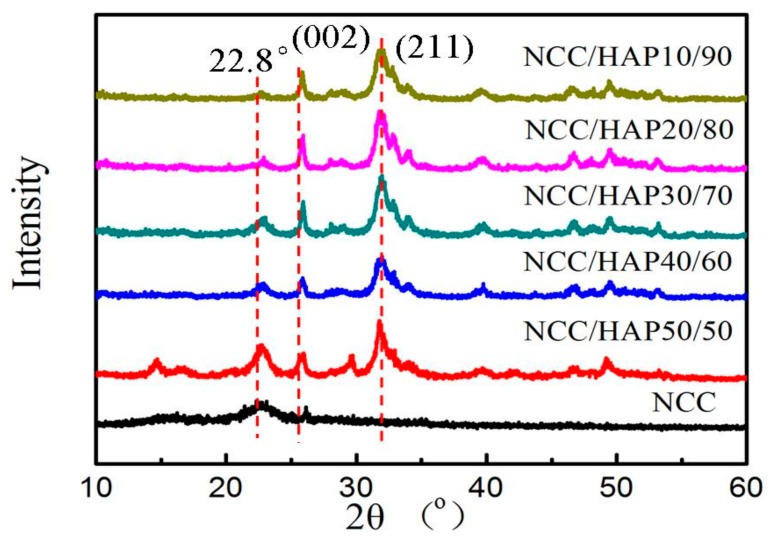
X-ray diffraction (XRD) of NCC, NCC/HAP50/50, NCC/HAP40/60, NCC/HAP30/70, NCC/HAP20/80 and NCC/HAP10/90.

The NCC and NCC/HAP structure was further characterized by XRD spectroscope and the results are shown in Figure 4. The XRD patterns exhibited diffraction peaks of NCC at 2θ values of 16.5°, 22.8°, attributed to plane (110), (200), which prove the cellulose I structure. With the increase of the HAP ratio, the diffraction peaks around 16.5°, and 22.8° gradually disappeared. For NCC/HAP, peaks at 2θ values of 25.5°, 32.1°, 32.5°, 40.0°, 47.1° correspond to (002), (210), (211), (310) and (222) lattice planes of HAP, respectively [47,48,49].

### 3.2. Performance of PLA/NCC-HAP Composite Films

#### 3.2.1. FT-IR of PLA/NCC-HAP

**Figure 5:**
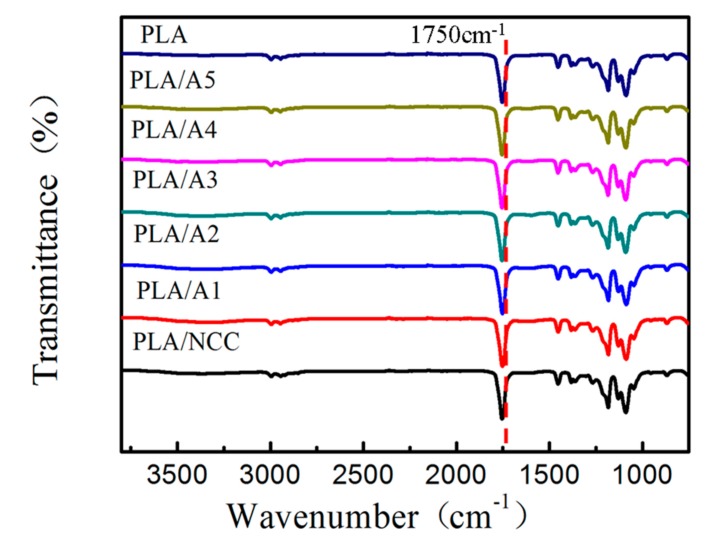
FTIR spectra of polylactic acid (PLA), PLA/NCC and PLA/NCC-HAP with different ratios of NCC to HAP.

In order to investigate the chemical structure of the composite, the FT-IR spectra were recorded for PLA, PLA/NCC and PLA/NCC-HAP with different ratios of NCC to HAP. The results are shown in Figure 5. There was no obvious difference among these samples. FT-IR spectra of pure PLA and modified PLA (PLA/NCC and PLA/NCC-HAP) display a similar characteristic peak at 1750 cm^−1^ which is attributed to carbonyl groups (C=O) from all the PLA-based nanocomposites [50].

#### 3.2.2. X-Ray Diffraction of PLA/NCC-HAP

**Figure 6:**
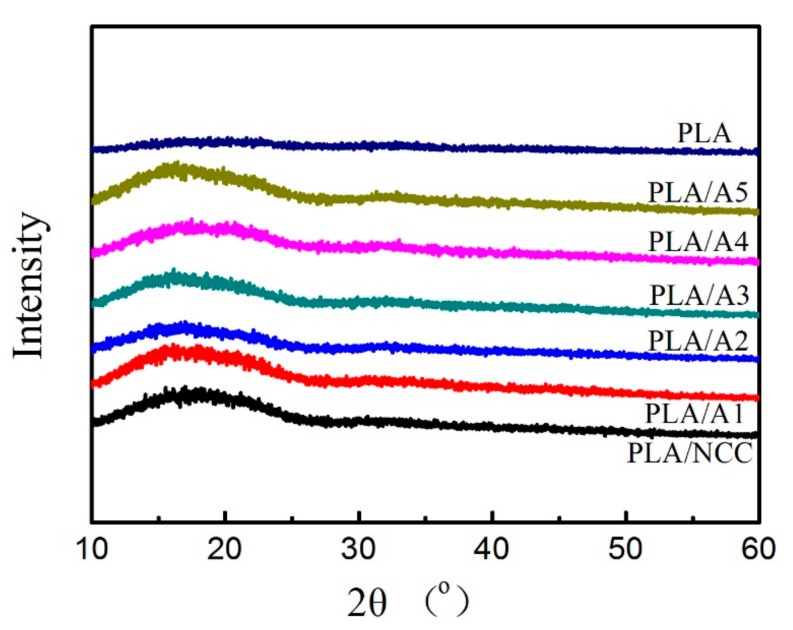
XRD characteristics PLA, PLA/NCC and PLA/NCC-HAP with different ratios of NCC to HAP.

XRD analysis of PLA, PLA/NCC and PLA/NCC-HAP was carried out in order to determine the crystalline structure. The results are given in Figure 6. Obviously, there are no significant diffraction peaks in pure PLA, due to its slow crystallization rate during molding, which resulted in an amorphous state. For the nanocomposites, the diffraction signals are masked by PLA owing to the low NCC and NCC/HAP content [51]. Moreover, there are no new uncharacteristic diffraction peaks found, which suggests that incorporation of NCC and NCC/HAP does not change the crystal structure of PLA [52].

#### 3.2.3. Mechanical Properties of PLA/NCC-HAP

**Figure 7:**
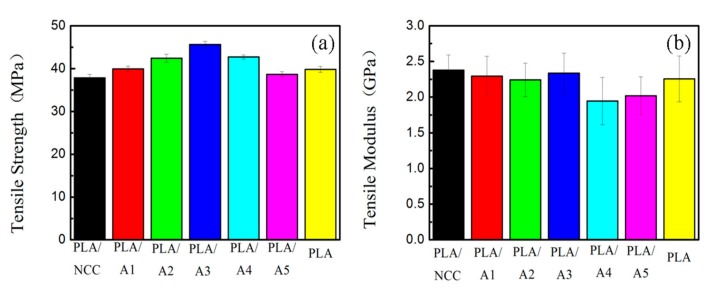
Mechanical properties of PLA/NCC-HAP films with different ratios of NCC to hydroxyapatite (HAP): (**a**) tensile strength and (**b**) tensile modulus.

The influence of NCC/HAP on the mechanical properties of the nanocomposite films was studied as displayed in Figure 7. The results show that PLA/A3 (NCC:HAP = 30/70) had the highest tensile strength of 45.6 MPa with a correspondingly high tensile modulus of 2.34 GPa. However, for PLA/NCC and PLA/A5 composite films, the tensile strengths decreased lower than that of pure PLA. This can be attributed to the limited compatibility of pristine PLA (it is hydrophobic) and NCC (it is hydrophilic). Additionally, when the ratio of NCC to HAP is too small (A5, NCC:HAP = 10/90), the composite films show poor compatibility. A suitable ratio of NCC/HAP improved the mechanical properties of the composite films, due to strong hydrogen bonding interaction at the interface, leading to good NCC/HAP dispersion in the PLA matrix [53].

**Figure 8:**
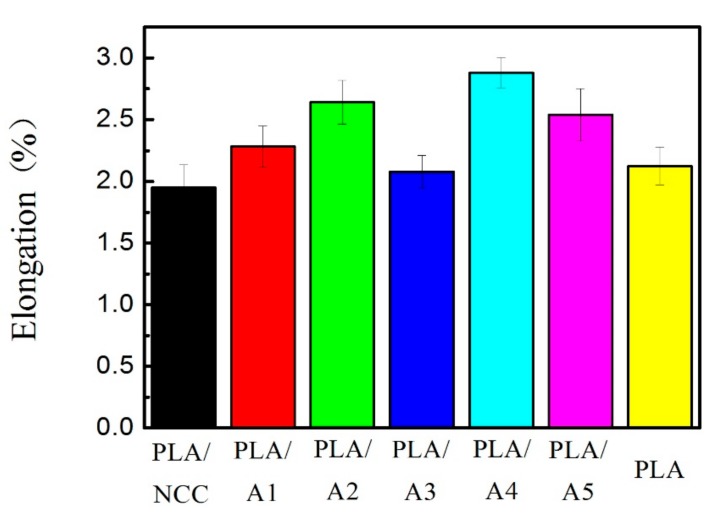
Elongation at break of PLA/NCC-HAP films with different ratios of NCC to HAP.

In order to explore the effect of NCC/HAP on the toughness of the nanocomposite, the elongation at break of samples is given in Figure 8. The results show that PLA/A3 (NCC:HAP = 30/70) had the maximum tensile strength and its elongation was only 2.08%. Combined with the above results of tensile modulus, the elongation at break of the samples has a negative correlation with the tensile modulus.

#### 3.2.4. SEM of PLA/NCC-HAP Fractured Surface

**Figure 9:**
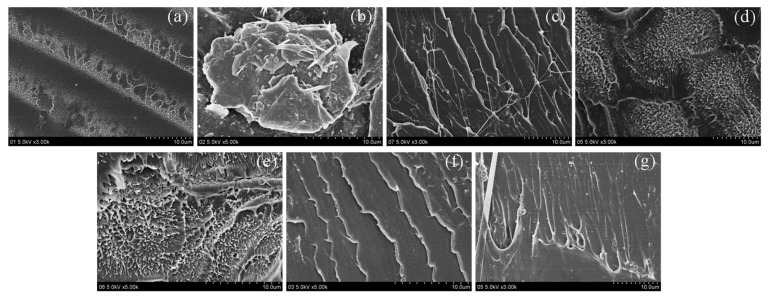
Scanning Electron Microscope (SEM) images of the fractured surfaces after tensile testing: (**a**) PLA, (**b**) PLA/NCC, (**c**) PLA/A1, (**d**) PLA/A2, (**e**) PLA/A3, (**f**) PLA/A4 and (**g**) PLA/A5.

In order to understand the relationship between the mechanical properties and the detailed structures, SEM micrographs of the fractured surfaces of the PLA/NCC-HAP composite films were carried out as presented in Figure 9. The pristine PLA fractured surface exhibits a smooth braided section, which indicates that the PLA is prone to brittle fracture. After the addition of NCC, the poor dispersion of PLA/NCC composites showed agglomeration and distinct cellulose rod-like structure. As the NCC/HAP added, the brittle fracture of PLA changes to a plastic fracture. When the ratio of NCC to HAP was 40/60 (A2) and 30/70 (A3), the fracture surface of the composite material showed a spurt-like surface with the voids caused by the extraction of cellulose nanorods, which indicated that the NCC-HAP interacted with the PLA matrix. It was also consistent with the increase in tensile strength [54,55].

**Figure 10:**
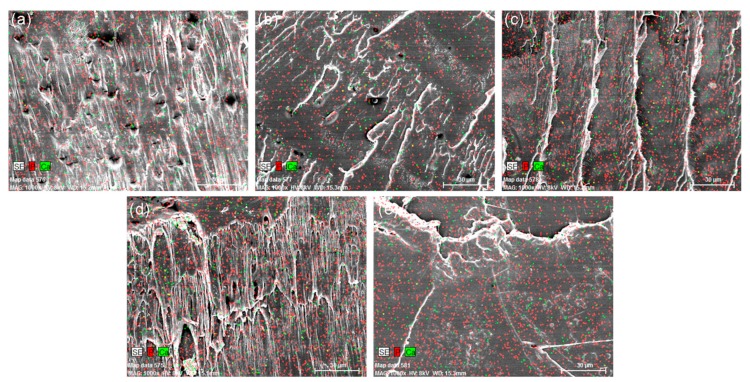
Energy Dispersive Spectrometer (EDS) of (**a**) PLA/A1, (**b**) PLA/A2, (**c**) PLA/A3, (**d**) PLA/A4, (**e**) PLA/A5.

In order to illustrate the distribution of main elements (Ca and P) at the cross section of the composites, the EDS results are shown in Figure 10. It can be seen that both Ca and P are uniformly distributed in the PLA/NCC-HAP composite films.

#### 3.2.5. DSC Characterization of PLA/NCC-HAP

**Figure 11:**
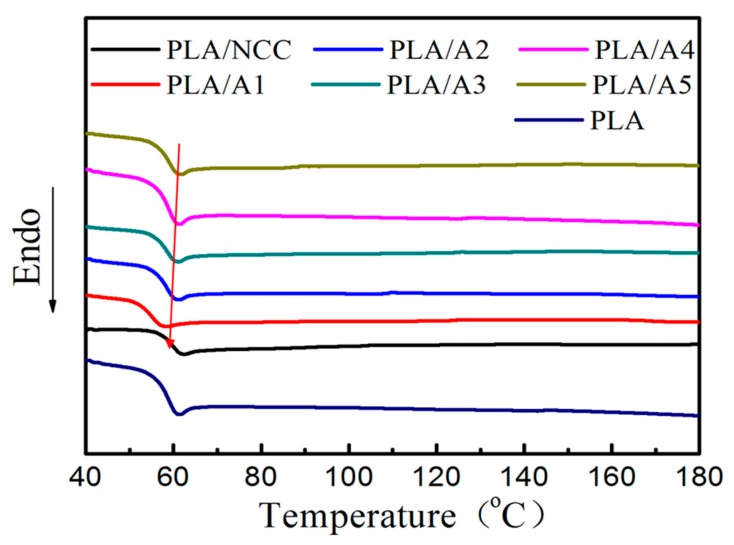
Differential scanning calorimetry (DSC) curves of PLA, PLA/NCC and PLA/NCC-HAP with different ratios of NCC to HAP.

The thermal behavior of PLA, PLA/NCC and PLA/NCC-HAP composites was determined by DSC, shown in Figure 11. The T_g_ of pure PLA is about 62.6 °C, and the addition of NCC/HAP shifted the T_g_ to the low temperature direction. The T_g_ of the PLA/NCC-HAP composite was slightly lower than 61 °C, indicating that NCC/HAP enhanced the compatibility with the PLA matrix. However, the T_g_ of PLA/NCC composites increased slightly, to about 63 °C. This indicates that the compatibility between NCC and PLA was not good, which was consistent with the above mechanical property results. Also, there was no crystalline peak and melting peak in DSC curves, suggesting that PLA, PLA/NCC and PLA/NCC-HAP composites in amorphous state have low crystallinity degrees, which is consistent with the observation claimed from XRD characterization results [56,57].

#### 3.2.6. TGA Characterization of PLA/NCC-HAP

**Figure 12:**
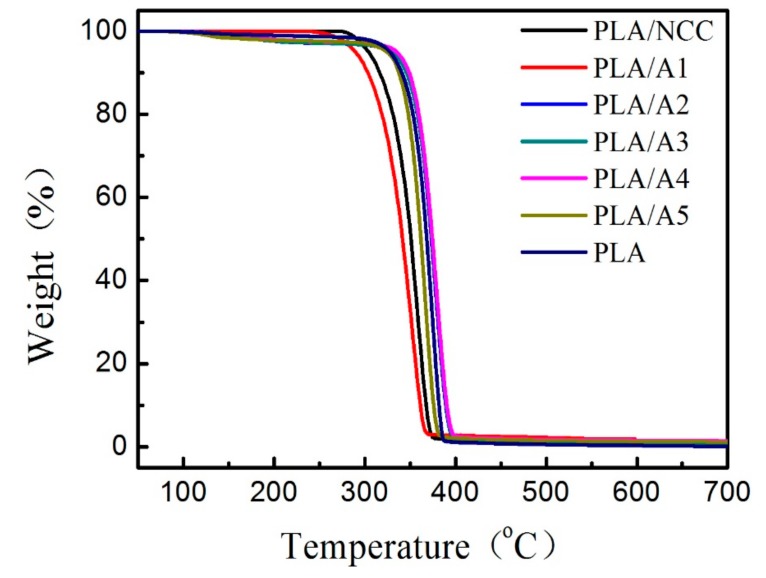
Thermal Gravimetric Analyzer (TGA) curves of PLA, PLA/NCC and PLA/NCC-HAP with different ratios of NCC to HAP.

To explore the thermal stability of the composite, the TGA curves for PLA, PLA/NCC and PLA/NCC-HAP composites were obtained as shown in Figure 12. The initial thermal decomposition temperature (T_10%_) of pure PLA thermal degradation was about 340.1 °C. There was no change in the thermal stability for PLA/NCC, PLA/A1, PLA/A5 composites. However, the addition of other ratios of NCC/HAP nanoparticles had a positive effect on the thermal stability of the composite. When the ratio of NCC to HAP was 40/60 (A2), 30/70 (A3) and 20/80 (A4), the T_10%_ of the composite was higher than 340.1 °C. This can be attributed to the interaction between NCC-HAP and PLA, hindering chain movement and inhibiting chain melting during the degradation process [58,59].

## 4. Conclusions

A series of NCC/HAP with different ratios were prepared to reinforce the PLA matrix. The thermal stabilities and mechanical properties of the obtained PLA/NCC-HAP composites were systematically investigated. In previous reports, the addition of inorganic nanoparticles resulted in a decrease in the tensile strength properties of the composite. Whilst here, the tensile strength, tensile modulus and thermal stability of the PLA/NCC-HAP composites significantly increased, especially for sample PLA/A3 (NCC/HAP at a ratio of 30/70). However, the addition of NCC/HAP does not accelerate the crystallization and neither improves the crystallinity degree of PLA. The above results show that NCC/HAP is a good reinforcing material for PLA. Since HAP is a bioceramic material with good osteoconductivity and osteoinductivity, the obtained PLA/NCC-HAP composite may be a promising medical repair or replacement material.

## Figures and Tables

**Table 1:** Chemical composition ratio for NCC/HAP preparation.

Group	NCC (g)	Ca(NO_3_)_2_·4H_2_O (g)	(NH_4_)_2_HPO_4_ (g)	Ratio
A1	0.5	1.175	0.394	50/50
A2	0.4	1.410	0.473	40/60
A3	0.3	1.646	0.552	30/70
A4	0.2	1.881	0.631	20/80
A5	0.1	2.117	0.710	10/90
